# Population bottlenecks and sexual recombination shape diatom microevolution

**DOI:** 10.1002/ece3.11464

**Published:** 2024-07-31

**Authors:** Bruno Hay Mele, Maria Valeria Ruggiero, Domenico D'Alelio

**Affiliations:** ^1^ Department of Biology University of Naples “Federico II” Naples Italy; ^2^ Stazione Zoologica Anton Dohrn Naples Italy; ^3^ National Biodiversity Future Center (NBFC) Palermo Italy

**Keywords:** diatoms, life cycles, microevolution, molecular evolution, sexual reproduction, SLiM

## Abstract

Diatoms are single‐celled organisms that contribute approximately 20% of the global primary production and play a crucial role in biogeochemical cycles and trophic chains. Despite their ecological importance, our knowledge of microevolution is limited. We developed a model using the SLiM evolutionary framework to address this knowledge gap. As a reference, we used the diatom *Pseudo‐nitzschia multistriata*, which has been extensively studied in the Gulf of Naples. Our model recapitulates what we observe in natural populations, with microevolutionary processes that occur annually during a three‐stage bloom phase. Interestingly, we found that non‐bloom phases allow the population to maintain sex‐generated diversity produced during blooms. This finding suggests that non‐bloom phases are critical to counteract bloom‐related pressures and mitigate genetic divergence at the species level. Moreover, our model showed that despite the consistent genetic differentiation during bloom phases, the population tends to return to pre‐bloom states. While our model is limited to neutral dynamics, our study provides valuable insights into diatoms' microevolution, paving the way to explore the ecological implications of the life history dynamics of these organisms.

## Graphical abstract

Diatoms are single‐celled organisms producing blooms crucial in biogeochemical cycles and trophic chains. We simulated neutral microevolutionary dynamics of synthetic diatom populations observing that non‐bloom phases are critical to counteract bloom‐related pressures and mitigate genetic divergence at the species level. Our work contributes to understanding microbial communities' roles in aquatic ecosystems and global biogeochemical cycles.

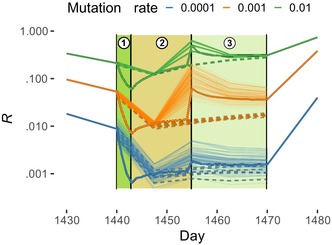

## 1 INTRODUCTION

Diatoms are among the most influential free‐living aquatic microbes. They are responsible for ~20% of the global primary production, drive biogeochemical cycles and represent the carbon entry point for trophic chains (Armbrust, 2009; D'Alelio et al., 2016). A typical demographic behaviour impacts these services: planktonic diatoms show sudden and periodical demographic explosions called blooms, driven by optimal conditions determined by environmental and biological factors (Behrenfeld et al., 2016; Cianelli et al., 2017; Wyatt, 2014).

Diatoms play an essential ecological role due to their vast diversity (Benoiston et al., 2017; Tréguer et al., 2018). Even within a single species, multiple lineages can exist simultaneously (Chen & Rynearson, 2016; Pérez‐Burillo et al., 2021), leading to a dynamic genetic structure (Ruggiero et al., 2022; Tesson et al., 2014) that promotes adaptation to changing environmental conditions (Godhe & Rynearson, 2017) and can even result in the formation of new species when lineages diverge in space or time (Percopo et al., 2022; Whittaker & Rynearson, 2017).

The diversity of diatoms results from their ‘diplontic’ life cycle (Montresor et al., 2016). Like other unicellular species, diatoms can reproduce asexually by binary fission and reach the density needed to overcome loss factors. Binary fission also promotes genetic variation through mitotic mutations during cell replication (Ruggiero et al., 2018). This long‐lasting diploid asexual phase can be seldom and briskly interrupted by sexual events that involve ephemeral haploid cells (gametes) (Montresor et al., 2016). In most diatom species, cell size decreases after each division and if the cells keep shrinking below a critical size threshold, they will die. Therefore, diatoms must undergo sex to restore their original cell size and prevent death (D'Alelio, Amato, Luedeking, & Montresor, 2009; Montresor et al., 2016). Diatom sex can be highly periodic, occurring with annual or even supra‐annual timing, probably due to the action of a biological clock (D'Alelio et al., 2010; Lewis, 1984).

Despite intraspecific diversity, the genetic structure of some diatom species appears stable over long‐term periods (Härnström et al., 2011) and some species show reproductive isolation in sympatry (Amato et al., 2007). This implies that no hybridisation occurs among cryptic species. Therefore, one can hypothesise that sex may promote species' ‘cohesion’ (sensu (Barker & Wilson, 2016)) by generating new ‘recombinant’ lineages while counteracting the divergence between ‘clones’ deriving from genetic mutations. However, our knowledge of the matter is subordinated to sampling diatom specimens in nature during transient blooms. Consequently, we lack a clear perception of diatom population genetics when they are not blooming due to the extreme rarity of cells during these ‘stationary’ phases. Data gained so far across time series indicate that diatom population composition (i.e., which lineages are present in a population and their frequency) can be similar before and after blooms, despite the strong genetic diversification occurring during a bloom (Ruggiero et al., 2018).

This study aims to expand our understanding of the impact of biological processes mentioned above, such as genetic recombination, mutation and population oscillations, on diatom population genetics across the bloom and non‐bloom phases. Such a step is needed to understand diatom microevolution better, i.e., the evolutionary change occurring over short periods within populations.

Using the SLiM evolutionary framework (Haller & Messer, 2019), we modelled the neutral evolutionary dynamics of in silico diatom populations and explored the interplay between bloom‐related demographic oscillations and genetic mutation and recombination. We simulated the time evolution of a diatom population by explicitly modelling (a) seasonal demographic pulses; (b) the regular alternation between asexual and sexual phases; (c) inheritable neutral mutations at microsatellite loci occurring during mitosis; (d) meiotic recombination between distinct lineages emerged from mitosis. We used the diatom *Pseudo‐nitzschia multistriata* as a reference, as its life cycle, population dynamics and genetics have been extensively studied in our reference marine site—i.e., the Gulf of Naples (Italy) during the Long‐Term Ecological Research MareChiara (D'Alelio et al., 2010; D'Alelio, Amato, Luedeking, & Montresor, 2009; Ruggiero et al., 2018). Our simulations were run under different mutation and recombination rates and model outputs were analysed using standard population genetic indices. In presenting our results, we will discuss the implications of the tangled microevolution of diatoms over species cohesion and population connectivity in time.

## 2 MATERIALS AND METHODS

### 2.1 Modelling context and rationale

We simulated neutral evolutionary dynamics of a synthetic population of diatoms based on the biological information available for *Pseudo‐nitzschia multistriata* (PM) from the Gulf of Naples (GoN) (D'Alelio et al., 2010; D'Alelio, Amato, Luedeking, & Montresor, 2009; Ruggiero et al., 2018; Tesson et al., 2011, 2013, 2014). We chose an individual‐based approach focused on the chromosome‐related processes of mutation and recombination. To follow evolutionary dynamics, we explicitly modelled microsatellites, herein defined as small genomic regions (loci) containing specific DNA motifs (commonly ranging from one to six base pairs in length) repeated between five and fifty times. At any specific moment, chromosomes can thus be described by the number of repeats of each microsatellite locus. We defined these patterns as multi‐locus genotypes (MLGs).

In our model, population reproduction depends on clonality and sex, while maximal population density depends on the system's carrying capacity. This choice allowed recreating the alternation between short‐term bloom periods, lasting 1 month, and long‐term stationary phases, lasting 11 months, characteristic of our reference diatom (D'Alelio et al., 2010; Lewis, 1984). For almost the whole duration of a simulated year, population reproduction is asexual and thus clonal, with a period‐dependent growth rate based on previous experimental and modelling data: cells divide twice a day during blooms and once every 5 days during stationary phases (D'Alelio et al., 2010).

We also imposed sex to occur every year, even though previous publications from GoN indicated that PM could undergo sex every 2 years (D'Alelio et al., 2010). This choice was motivated by biomolecular data suggesting that sex in PM is more frequent than previously hypothesised. Besides, the biennial cycle appeared to break after 10 years of sample observation (D'Alelio et al., 2010; Ruggiero et al., 2018). We programmed sex to occur on the day at the middle of each annual bloom, i.e., at the end of a real bloom's exponential phase, when part of the population is known to produce gametes (Lewis, 1984).

During clonal reproduction, the number of microsatellites repeats within each locus can change with a fixed probability (mutation rate, *mut_rate*); during sex, occurring with a fixed number of cells in the population (defined by the *sex_rate* parameter), recombination events will arise through crossing‐over between adjacent bases. The chance of crossing‐over is regulated by the recombination rate (*rec_rate*), defined as the probability of breakpoint formation (i.e., the point where the homologous chromosomes can break and recombine). It is worth noting that while recombination breakpoints occur randomly on the chromosome, they will never interfere with microsatellites (our genetic marker of choice), i.e., they never break the tandem repeat.

Simulations were built using the SLiM framework for chromosome‐based models (https://messerlab.org/slim/, https://doi.org/10.1093/molbev/msy228). SLiM permits us to define the structure and properties of chromosomes and then to follow how chromosomes change in time based on the population dynamics imposed. Here, we defined a chromosome with five microsatellites at coordinates randomly assigned during model start‐up. We used the nonWF model type (i.e., non‐Wright‐Fisher; Haller & Messer, 2019) to define explicit mating and to have more control over offspring generation (i.e., chromosomal recombination). Loss terms (deaths of individuals) are not explicitly modelled but emerge during the sampling steps. More specifically, at every epoch (day) and after reproduction the system takes a sample of size equal to the carrying capacity. This reproduces neutral selection and contributes to the emergence of the post‐bloom bottlenecks.

### 2.2 Model parameterization

Our model was parameterised by *mut_rate*, *rec_rate* and *sex* *_rate*. In PM, the mitotic mutation rate of microsatellites was reported to be 0.003 (Tesson et al., 2013). Here, *mut_rate*, i.e., the chance of variation in microsatellite repeats, ranges from 10^−4^ to 10^−2^. As for *rec_rate*, meiotic recombination rates in diatoms are unknown, at least based on our knowledge. Therefore, we referred to information available for other free‐living protists (Hasan & Ness, 2020). To minimise the bias associated with the selection of an incorrect order of magnitude and to accurately simulate the interplay between recombination and mutation, we performed simulations at four different recombination regimes ([1 × 10^−8^, 1 × 10^−4^, 10^−2^]). Finally, we set *sex_rate* at three different values (20%, 35%, 50%) considering experimental in‐vivo and in‐situ evidence (D'Alelio, Amato, Luedeking, & Montresor, 2009; Sarno et al., 2010). The system's carrying capacity was set to switch between 10^2^ and 10^5^ individuals to simulate stationary phases and blooms, respectively, based on in‐situ observations for PM and similar diatoms (D'Alelio et al., 2010; D'Alelio, Amato, Kooistra, et al., 2009; D'Alelio, Amato, Luedeking, & Montresor, 2009; Godhe et al., 2013; Kim et al., 2020; Lewis, 1984; Ruggiero et al., 2018).

We simulated 36 (3 × 4 × 3) combinations of *mut_rate*, *rec_rate* and *sex rates* plus three different mutation rate regimens for the obligated clonal population (where *sex_rate* = 0) for a total of 39 different conditions. Since mutation and recombination are random processes at their core, we performed six simulation runs for each condition. This strategy allowed us to capture the average dynamics of genetic diversification. Each of those runs started from the same input population.

To simulate mature populations, we performed an 11‐year ‘burnout’ during which populations grow unmonitored. After that period, we sampled populations for 5 years to obtain an estimate of parameters representative of microevolutionary dynamics. To capture general trends, we collected population genetics status every 2 months, outside blooms and once every week, inside blooms, for all combinations. To detail the behaviour of sexual populations, we ran one additional simulation at 35% sex for all mutation rate values sampling at higher temporal resolution (every 10 days, outside blooms and every day, within blooms; parameters: *rec_rate* = 10^−6^; *sex_rate* = 0.35). The different runs were maintained identically for the first days to ensure they were replicate tests.

### 2.3 Population genetics metrics

To assess the genetic status of each synthetic population, we calculated the following indexes at each sampling event: (i) allele and multilocus genotype count (*A* and *G*, respectively); (ii) heterozygosity (*H*) and (iii) genotypic diversity (*R*), following (Dorken & Eckert, 2001). We compared the index distribution between pre‐ and post‐bloom days and within/outside the bloom for all the parameter combinations. We also explored the allelic composition on each day as the presence/absence of alleles and calculated the binomial distance (Anderson & Millar, 2004) between all days for all parameter combinations. A scatter plot and a Principal Component Analysis (PCA) were produced from the genetic indices' matrix and a non‐metric dimensional scaling (nMDS) was produced from the allele presence/absence matrix.

### 2.4 Coding

Our model is available as a commented .slim source on https://github.com/bhym/diatoms_microevo. We used the R software environment for data aggregation and analysis. Model outputs were imported and tidied using dplyr and tidyr packages from the tidyverse collection (https://www.tidyverse.org/). All visualisations but the ordinations (nMDS and PCA) were performed using ggplot2 from the same collection and aggregated using the patchwork package (https://CRAN.R‐project.org/package=patchwork). PCA of the index matrix was performed using the singular value decomposition (prcomp) method and visualised using the ordiplot function from vegan (https://CRAN.R‐project.org/package=vegan). The ordination analysis on population matrices was performed using the vegan package. A binomial distance matrix was calculated using the vegdist function with the “binomial” method and the metaMDS function with default parameters was used to compute the nMDS. We evaluated the nMDS using the stressplot function and visualised the nMDS using ordiplot. Code for figure generation is available on https://github.com/bhym/diatoms_microevo.

## 3 RESULTS AND DISCUSSION

**FIGURE 1:**
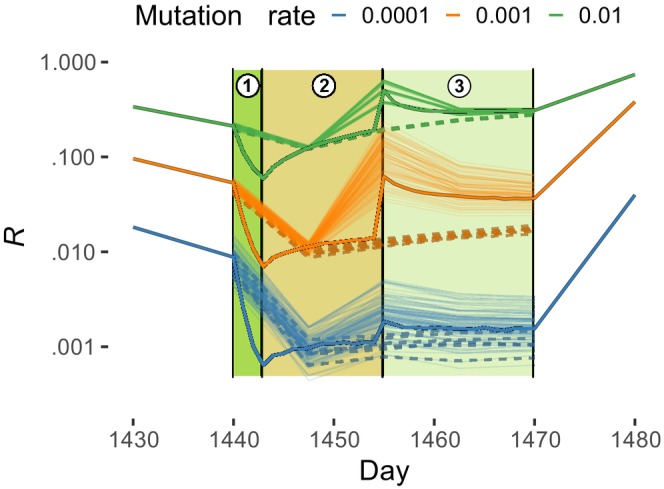
The plot of genetic diversity (*R*) within‐bloom dynamics (using the time range of the fourth bloom) using Mutation rate for colour code and line type to depict asexual (dashed) and sexual (solid) simulations. Each point is the index's value for a specific repetition/combination of parameters at a specific time. Lines hint at the index trajectories in times per repetition/combination of parameters. Thicker lines are simulations with higher temporal resolution; coloured and indexed bands correspond to the bloom signature stages: *stage one*: start of the bloom—diversity drop; *stage two*: blooming and sex—diversity accumulation and spike; *stage three*: the second half of the bloom—diversity decay and plateau. The entire simulation dynamic is available in Figure S1.

**FIGURE 2:**
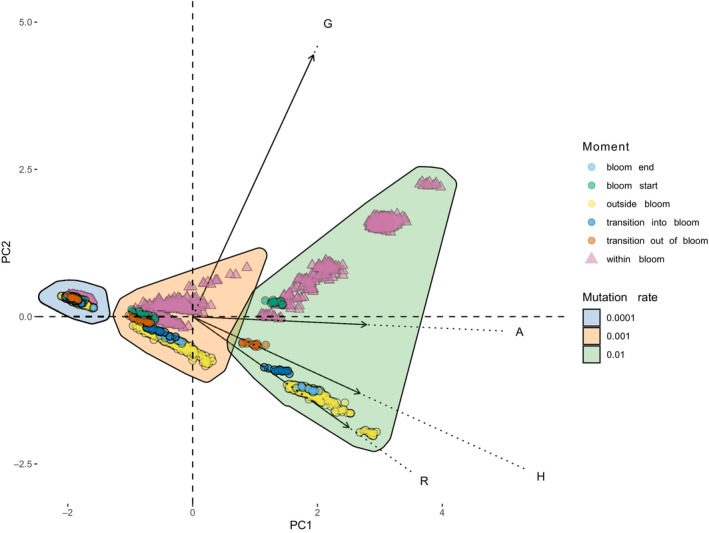
Principal component analysis of the index matrix (i.e., a table where rows are days and columns are index values for each repetition/combination of parameters; indexes are allele count, multilocus genotype count, heterozygosity and genetic diversity). Polygons mark different mutation rates (from left to right: 1 × 10^−4^, 1 × 10^−3^, 1 × 10^−2^). Arrows refer to the contribution of each index (*G*—Genotype count, *A*—Allele count, *H*—Heterozygosity, *R*—Genetic diversity) to the Object ordination (Parameter rates: *sex_rate* = 0.2, *rec_rate* = *mut_rate* = 1 × 10^−4^).

**FIGURE 3:**
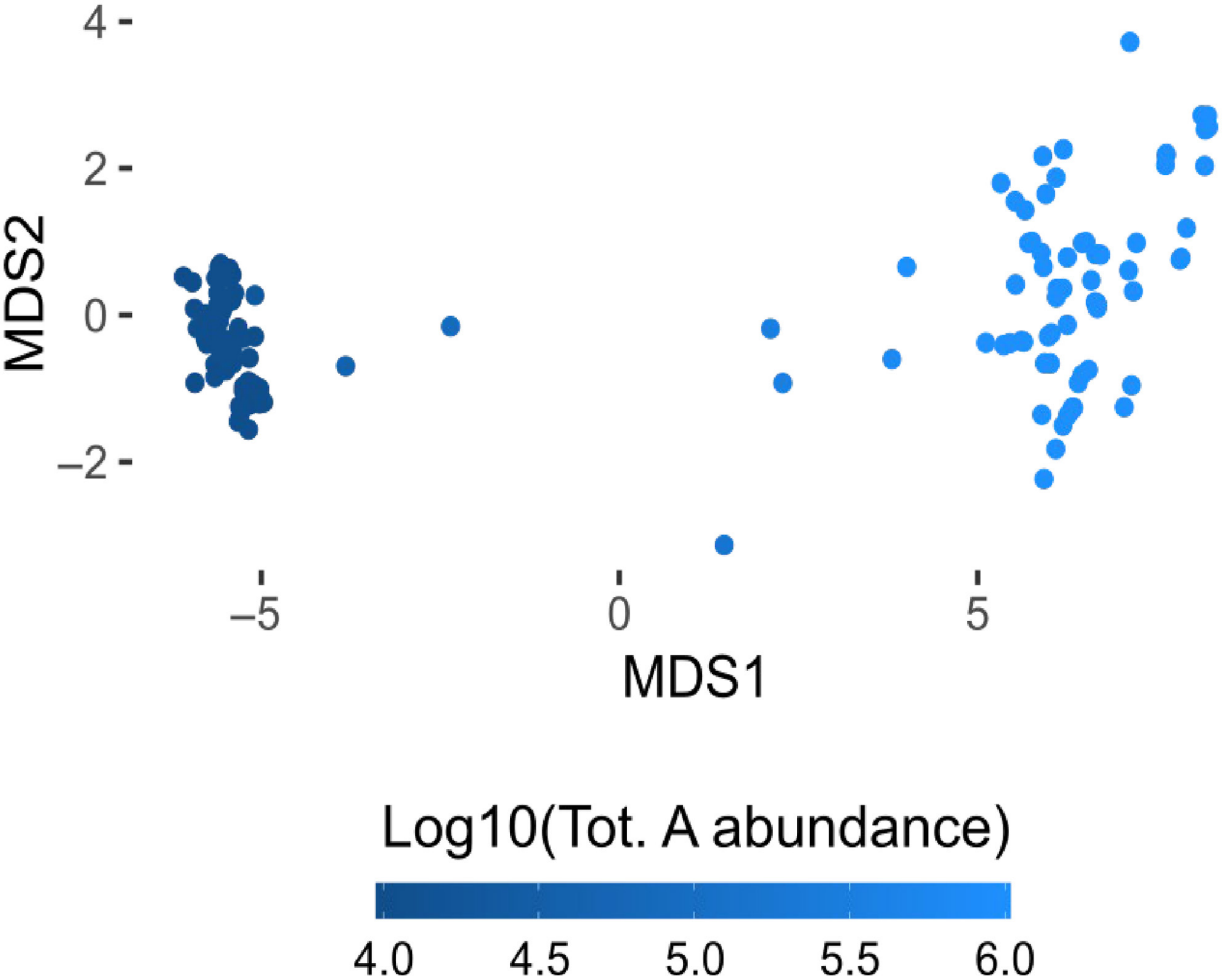
Ordination panel for the simulations having the following parameter combination: *rec_rate* =1 × 10^−4^, *mut_rate* = 1 × 10^−4^, *sex_rate* = 0.2. Points represent the genetic composition of the population each day; the colour scale marks abundance differences in time.

**FIGURE 4:**
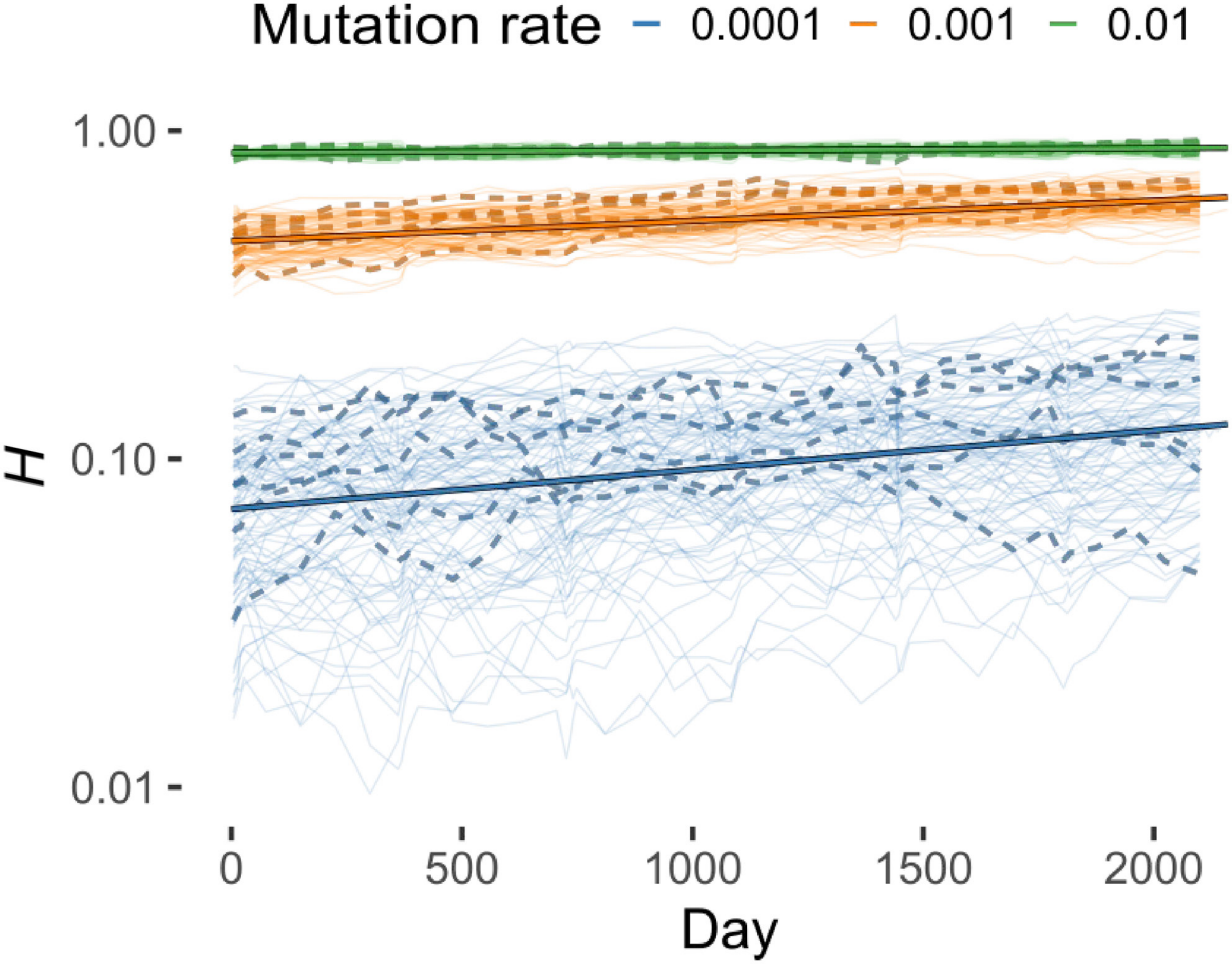
The plot of heterozygosity (*H*) measured across the entire simulation duration for all repetitions of all parameter combinations. The mutation rate is encoded as colour as per legend; line type depicts asexual (dashed) and sexual (solid) simulations. Thicker lines are linear fits.

The complex and non‐linear population dynamics of diatoms arise from their diplontic life cycle and seasonal blooms. While the former generates genetic diversity through mutation and recombination, seasonal blooms modify diversity through demographic fluctuations. In our model, clonality and sexual reproduction drive changes in the genetic makeup of individual diatoms. These changes propagate at the population level, impacting overall genetic diversity and structure (Figures 1, 2, 3, 4).

Our study aimed to model the evolution of diatom populations under specific biological (i.e., periodic sex) and ecological (i.e., time‐dependent carrying capacity) constraints. Specifically, we investigated how changes in mutation and recombination rates affect population structure under a constrained demographic regime. Our results showed that microevolutionary processes are strongly influenced by yearly dynamics typical of diatoms (i.e., blooms), even under varying diversity production rates. Interestingly, regardless of the magnitude, the bloom phase exhibited a three‐stage signature, like the one characterising data gathered from field observations of our model species (Ruggiero et al., 2018) (see Figure 1 for the behaviour of the *R* metric; other indexes display the same trend). The emergence of a known biological signature is proof that our model recapitulates a characteristic diatom genetic dynamics.

### 3.1 Mutation and recombination interplay in diatom microevolution

While asexual reproduction introduces mitotic mutations in diatoms, periodic sexual reproduction recombines the genetic makeup of populations, as observed in multiple microorganisms (D'Alelio, Amato, Kooistra, et al., 2009; Godhe et al., 2013; Kim et al., 2020). Our results show that these two forces interact to modulate genetic diversification, as evidenced by the time course of genotypic diversity across a bloom. This finding supports the observation that multiple distinct lineages can coexist within a single bloom of a diatom species (Chen & Rynearson, 2016; Pérez‐Burillo et al., 2021).

*R* shows a significant decrease during the early stage of the bloom, as population density increases (and will remain high for the entire duration of the bloom) while the number of multilocus genotypes (MLGs) remains constant (stage one, Figure 1). As the bloom progresses, *R* slowly increases due to the incorporation of new MLGs generated through mutation, recombination or both (stage two). Comparison of asexual and sexual model runs (Figure 1, dashed vs solid lines) indicates a sharp increase in *R* following a sexual event (the spike separating phase two from three), which is positively correlated with the number of sexually active cells. Finally, during the second half of the bloom, *R* slowly decays (stage three) due to diversity erosion caused by genetic drift. Our findings suggest that, as seen in other similar organisms tested in laboratory conditions, sexual reproduction can ‘release the speed limit of evolution’ (Colegrave, 2002).

In our in silico population, the gain of genetic diversity during the second part of the bloom period, which followed sexual reproduction, was smoothed by the random removal of individuals and MLGs. Nonetheless, a further increase in *R* was observed due to the bottleneck caused by the end of the bloom, which led to a contraction of the carrying capacity. This bottleneck impacted the clonal subpopulations representing the most abundant MLGs, resulting in a shift of the *G/N* ratio in favour of the number of genotypes (*G*). Such bottlenecks and their effects were also observed in natural populations of PM (Ruggiero et al., 2018).

### 3.2 Demographic processes and genetic discontinuity

Planktonic diatoms undergo periodic blooms driven by physical, chemical and biological factors. However, these events are short‐lived and after the population bursts, the species often become undetectable (Behrenfeld et al., 2016; Cianelli et al., 2017; Wyatt, 2014). This dynamic may provide an advantage by reducing competition between closely related species, which tend to have desynchronised boom‐bust cycles (Doebeli et al., 2021). Such a hypothesis could explain why different conspecific diatom lineages preferentially produce blooms at different environmental conditions. Speciation occurs when lineages separate in time, space or both (Percopo et al., 2022; Whittaker & Rynearson, 2017).

Figure 2 displays the ordination of daily population states in the space of all considered metrics (*H*, *R*, *A*, *G*). On the one hand, the number of MLGs (*G*) varied during blooms (*G* vector), driving these population states away from pre‐bloom conditions. On the other hand, heterozygosity (*H*), genotypic diversity (*R*) and allele counts (*A*) tended to vary outside of blooms. This relationship suggests that while bloom phases promote population diversification, non‐bloom phases enable the population to retain a significant fraction of the diversity generated through sex during a bloom. In other words, hybrid lineages produced by recombination are stored during non‐bloom phases, counteracting bloom‐related pressures and mitigating genetic divergence at the species level.

### 3.3 Population bottlenecks, sexual recombination and species cohesion

Although our model is limited to neutral evolutionary dynamics, it could be used to explore the role of sex and demographic processes in promoting species cohesion (Barker & Wilson, 2016). To investigate this, we performed a simulation with identical mutation and recombination rates (10^−4^) where 20% of individuals participated in sexual events, like what is observed in natural *Pseudo‐nitzschia* spp. (D'Alelio, Amato, Luedeking, & Montresor, 2009; Sarno et al., 2010). We evaluated the distance between populations during the simulation and projected the distance matrix onto a 2D space using nMDS (Figure 3).

The visualisation suggests that, despite consistent genetic differentiation occurring during bloom phases, the population tends to return to an initial state during non‐bloom periods. However, this behaviour could not be solely explained by the boom‐bust demography. Indeed, the act of sex was also detectable when considering the time course of heterozygosity, which slightly but constantly increased over time (Figure 4). The increase can be due to the sample effect: during the bottleneck phase, clonal lines that contribute less to heterozygosity are preferentially removed.

### 3.4 Ecological and evolutionary implications

Diatoms are ecologically relevant aquatic microbes (Armbrust, 2009; D'Alelio et al., 2016), they undergo frequent and massive blooms in the ocean regulated by the tangled interplay of physical, chemical and biological factors, such as life‐cycle shift (Behrenfeld et al., 2016; Benoiston et al., 2017; Cianelli et al., 2017; Tréguer et al., 2018; Wyatt, 2014). Multiple lineages co‐exist in a single species (Chen & Rynearson, 2016; Pérez‐Burillo et al., 2021) and from such a genetic melting pot, new species can emerge when lineages diverge in space or time (Percopo et al., 2022; Whittaker & Rynearson, 2017). Such a somewhat perpetual genetic diversification may be promoting adaptation to the intermittent environmental conditions found in the coastal zones (Godhe & Rynearson, 2017). However, our study demonstrates that clonal lineages do not necessarily diverge in time due to the combined action of demographic oscillations and life‐cycle processes like clonality and sex, in a way that species can reinforce their genetic pool but keep their genetic cohesion in time.

Based on our model simulation, the considerably high‐clonal (i.e. mutation‐based) diversity generated during a diatom bloom is removed more frequently than sexual (i.e. recombination‐based) diversity produced during the single sexual event occurring at the higher population density occurring during a bloom. This is especially true during the post‐bloom bottlenecks generated by the induced collapse of the carrying capacity. While mutation causes the accumulation of alleles, and thus, an increase in genetic richness, genetic drift acts as a density‐dependent force that removes multilocus genotypes based on their abundance.

Our model observation nicely fits the picture emerging from in‐situ observations carried out for the bloom‐forming diatom *P. multistriata* (Ruggiero et al., 2018, 2024), whose population genetics was investigated in the Gulf of Naples for more than a decade, thus providing the present study with background knowledge and numerical information to parameterise our model. We can hypothesise that the population‐genetics dynamics described in this study are proper for *P. multistriata* only but cannot exclude that it may be shared with many other diatoms since at least another distantly related diatom showed a stable genetic structure over long term (Härnström et al., 2011). Nonetheless, studying the diatom microevolution over long term is a challenging task and further efforts to this end are worth taking, especially in the frame of Long‐Term Ecological Research studies, providing resources to isolate and characterise genetically multiple strains of these microscopic creatures.

In conclusion, our modelling study paves the way for new investigations coupling in‐situ and in‐silico approaches, such as population genetics and demographic models, to fully understand the mechanisms behind the success of diatoms in the global ocean.

## AUTHOR CONTRIBUTIONS

**Bruno Hay Mele:** Conceptualization (equal); data curation (equal); formal analysis (equal); investigation (equal); methodology (equal); software (equal); validation (equal); visualization (equal); writing – original draft (equal); writing – review and editing (equal). **Maria Valeria Ruggiero:** Conceptualization (equal); data curation (equal); formal analysis (equal); investigation (equal); methodology (equal); software (equal); supervision (equal); validation (equal); visualization (equal); writing – original draft (equal); writing – review and editing (equal). **Domenico D'Alelio:** Conceptualization (equal); funding acquisition (equal); investigation (equal); methodology (equal); supervision (equal); writing – original draft (equal); writing – review and editing (equal).

## CONFLICT OF INTEREST STATEMENT

The authors declare no conflict of interest.

## Supporting information


Figure S1

## Data Availability

Coding and data are available at https://github.com/bhym/diatoms_microevo.
