# Supplementary material for: Population bottlenecks and sexual recombination shape diatom microevolution
Source: Ecol Evol. 2024 Jul 31;14(8):e11464. doi: 10.1002/ece3.11464 (PMC11289787; doi:10.1002/ece3.11464)
Supplement: Supplementary file 1 — Figure S1 [file ECE3-14-e11464-s001.docx]

# Supplementary Figure


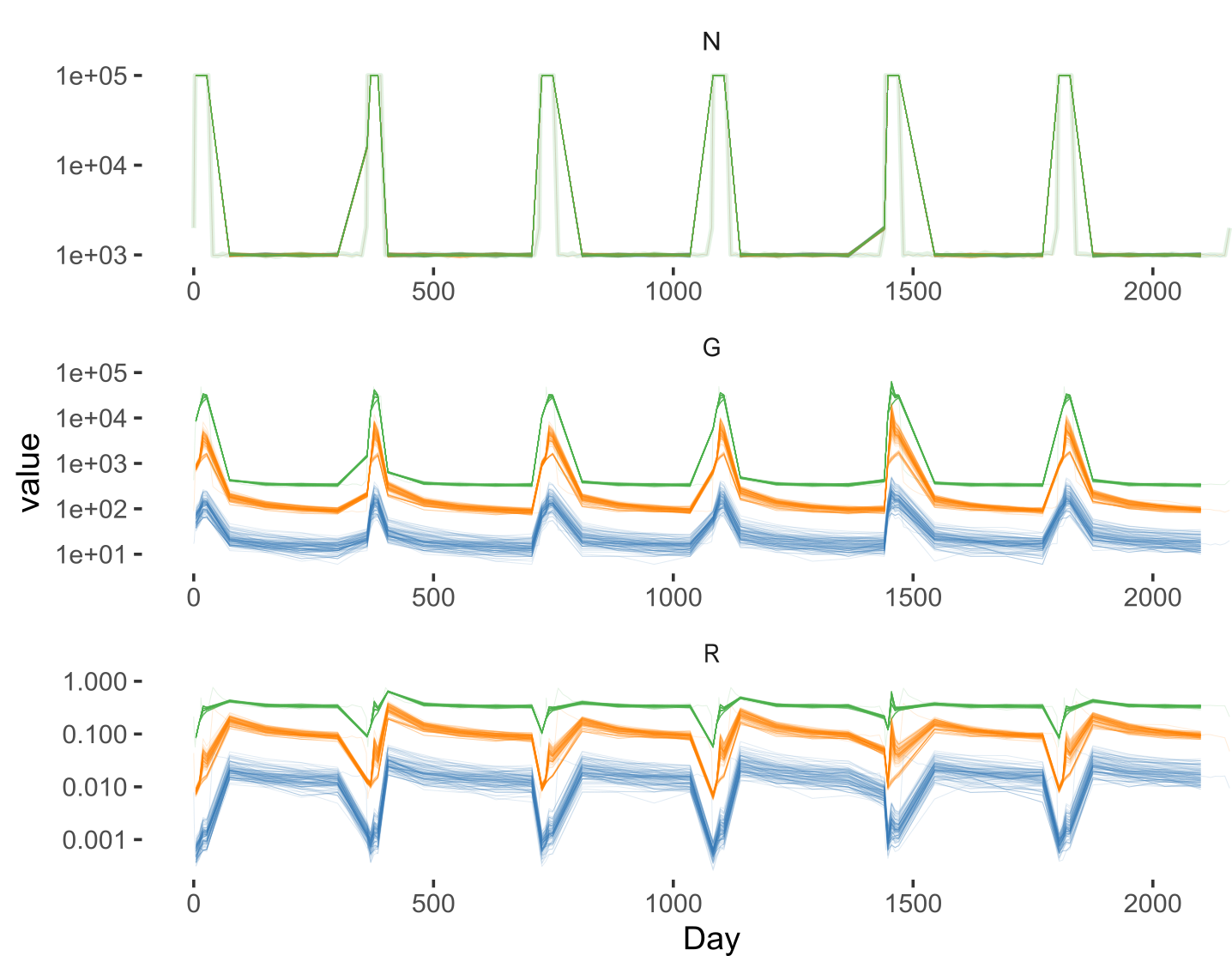


Fig S1. Time evolution for R, G, and N for all the combinations of parameters. Colour marks mutation rate as per Fig 1. Demographic dynamics (N row) are displayed for extended populations as thin lines (cfr. Model parametrization section in the main text).
